# Supplementary material for: AAV-CRISPR/Cas9 Gene Editing Preserves Long-Term Vision in the P23H Rat Model of Autosomal Dominant Retinitis Pigmentosa
Source: Pharmaceutics. 2022 Apr 9;14(4):824. doi: 10.3390/pharmaceutics14040824 (PMC9026811; doi:10.3390/pharmaceutics14040824)
Supplement: Supplementary file 1 [file pharmaceutics-14-00824-s001.zip › pharmaceutics-1644713-proofed-supplement.pdf]

pharmaceutics

MDPI

Supplementary Materials: AAV-CRISPR/Cas9 Gene Editing Preserves Long-Term Vision in the P23H Rat Model of Autosomal Dominant Retinitis Pigmentosa

Saba Shahin, Hui Xu, Bin Lu, Augustus Mercado, Melissa K Jones, Benjamin Bakondi and Shaomei Wang

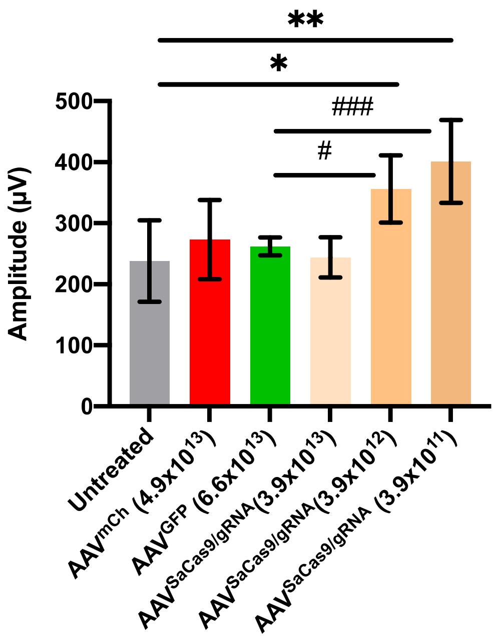

Figure S1. AAV controls. To assess AAV toxicity and determine optimal dose, P15 P23H/SD rats received a single unilateral trans-scleral subretinal injection of 2µl AAV at concentrations of 3.9 × 10^{13} GC/ml (244 ± 33µV, *n* = 14), 3.9 × 10^{12} (356 ± 55µV, *n* = 3), or 3.9 × 10^{11} (401 ± 68µV, *n* = 3) (orange bars), and control rats received either AAV8.CMV-mCherry (AAVmCh 4.9 × 10^{13} GC/ml, red bar), or AAV8.CMV-GFP (AAVGFP 6.6 × 10^{13} GC/ml, green bar), or left untreated (untreated controls: grey bar). ERG at P90 showed that retinal perturbation from AAV controls significantly reduced visual function, whereas AAV containing SaCas9/gRNA showed improved visual function and had no deleterious effect compared to sham-operated eyes. Dosages are shown as genome copies (GC) per microliter. Data represents the mean ± SEM. $^{\#}p \leq 0.05$, $^{\#\#\#}p \leq 0.01$; * $p \leq 0.05$, **$p \leq 0.001$.

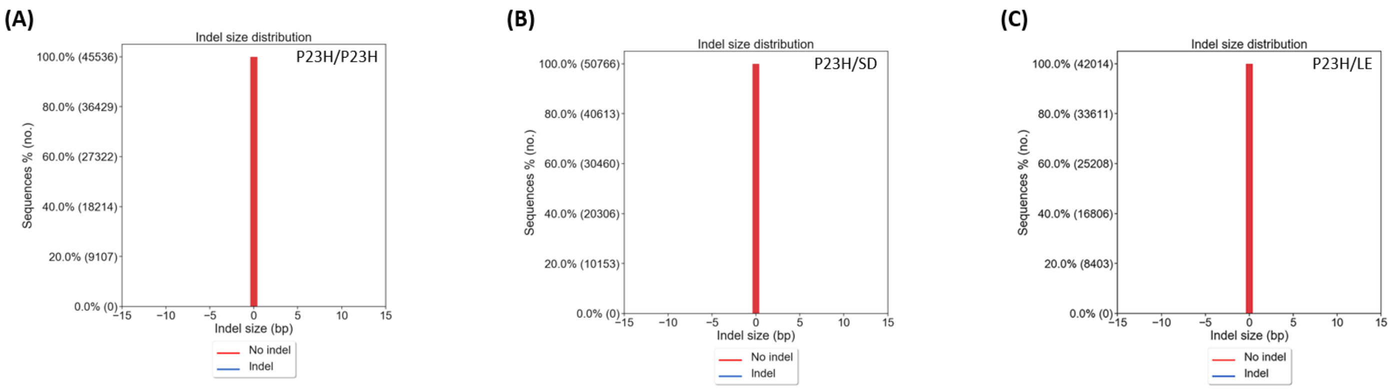

Figure S2. Indel profile of untreated controls: Representative graphs showing the frequencies and distributions of indels in untreated P23H [P23H/P23H (**A**), P23H/SD (**B**) and P23H/LE (**C**)] rats. No insertion and/or deletion were detected at predicted cleavage site of the targeted region.

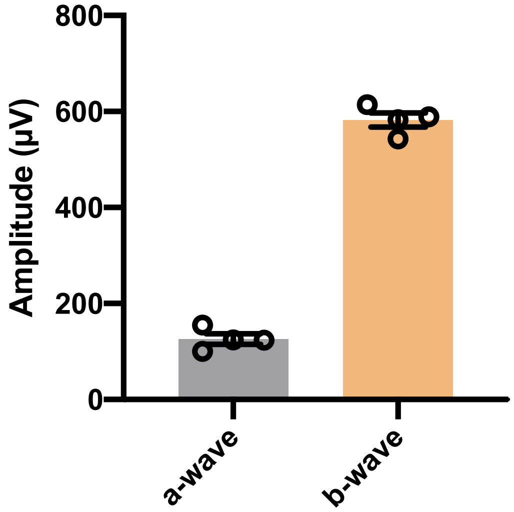

Figure S3. Electroretinography (ERG) of wild type Long Evans (LE) rats: Four adult LE rats, both male and female were dark-adapted overnight. ERG response amplitudes to full field light stimulation were recorded. ERG showed no change in a-wave (125.96 ± 11.26 µV) and b-wave (582.26 ± 14.69 µV) in these four LE rats of different ages (P124, P154 and P284). Data represents the mean ± SEM (n = 4).

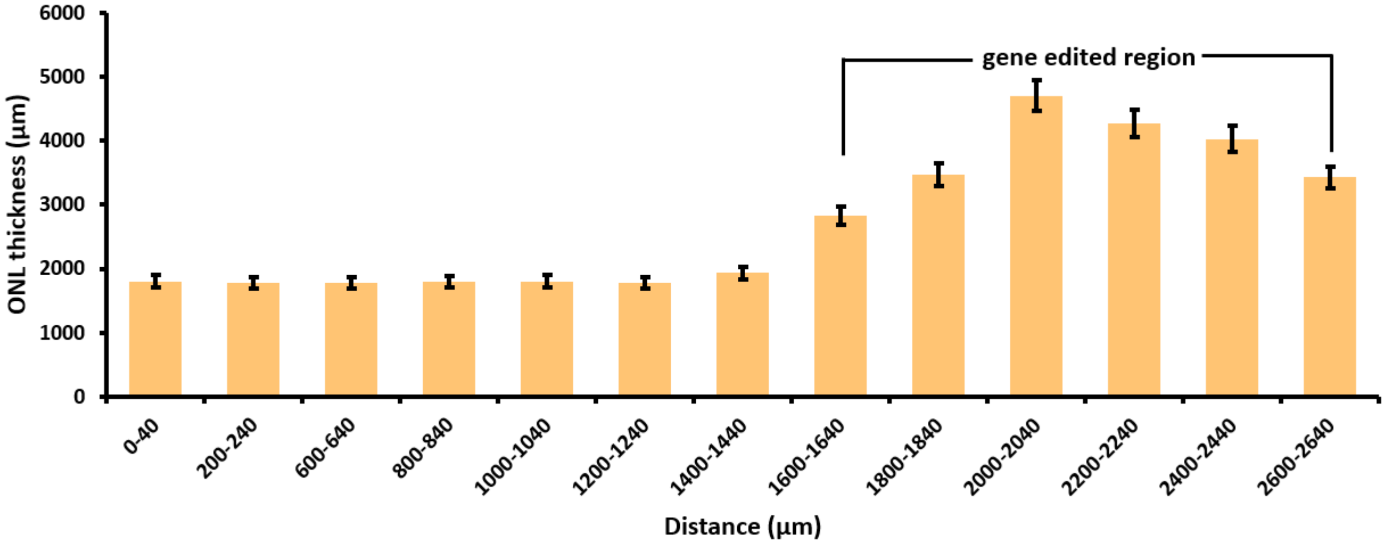

Figure S4. Quantification of ONL thickness along its rostral-caudal axis up to 2640 µm in the AAV-treated retina: ONL rescue was documented in the gene edited region (from 1600 µm to 2640 µm) that clearly demonstrates photoreceptor rescue in this gene edited region of the AAV-treated retina.

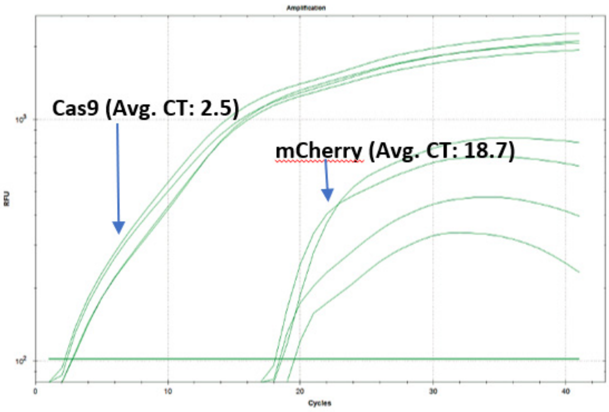

	Ct values	avg.Ct	deltaCt	RQ
CMV-mCherry1	19.58	18.695	16.1525	72,843
CMV-mCherry2	18.09			
CMV-mCherry3	18.39			
CMV-mCherry4	18.72			
ntc	0			
mOp500-SaCas1	2.85	2.5425		
mOp500-SaCas2	2.79			
mOp500-SaCas3	2.34			
mOp500-SaCas4	2.19			
ntc	0			

Figure S5. Assessment of AAV packaging efficiency. Real-time PCR amplification showing AAV packaging frequency of mCherry compared with SaCas9 transcripts. Cycle Threshold (CT) values are shown for amplified vector regions spanning the mOp500 promoter and SaCas9 (avg. CT: 2.5), or CMV promoter and mCherry transcripts (avg. CT: 18.7). Relative quantification (ΔCT: 16.15) indicated that the SaCas9 transcript was 7.2 × 10^4–fold more abundant than mCherry. RT-PCR was performed in quadruplicate using SYBR-green detection. Primer sequences are as follows: CMV-mCherry: Forward primer GTAACAACTCCGCCCCATT, Reverse primer: ATGGCCATGTTATCCTCCTC; mOp500-SaCas9: Forward primer GGGTCAGTGCCTGGAGTT, Reverse primer: ATACCGACCTTCCGCTTCT.

Table S1. Predicted on- and off-targets sites for gRNA.

	Target Sequence	PAM	Mis-match	Fwd Priming Site	Rev Priming Site
OFF-T1	TGGaCCCAAAGAaacAG-cAGCCA	GGGAGT	5	GGAAAACACAGTGC-TACAGTC	TCAGGAA-GAGGTTTCATGGT
OFF-T2	TaGGCCCAAA-tAttAtGTAGCCA	GTGAGT	5	TTCTCTCAACGGG-GATTGAC	GACATTAGGAGCCAC-CTACC
OFF-T3	TGtGCaCAAAtACacAG-TAGCCA	AA-GAGT	5	GAGCTG-TACACAGGTCTAGC	GAAGGACAGCAGA-GAGCTAA
ON-T	TGGGCCCAAAGAC-GAAGTAGCCA	TGGAGT	0	ACAGAGGGCCCCAATTTTTA	CCCAG-TCTCTCTGCTCATAC

Table S2. Primer sequences used for qPCR.

Gene	Sense (5'-3')	Antisense (5'-3')	Accession Number
r-Atf6	ATGGAGTCGCCTTTTAGTCC	CTGTACCGACTCAGGGAGGG	NM_001107196.1
r-Atg5	ACCTCGGTTTGGCTTGGTTG	AGTATGGCTCTGCTTCTCGTT	NM_001014250.1
r-Atg7	AGCCTGTTCATCCAAAGTTCT	CTGTGGTTGCTCAGACGGT	NM_001012097.1
r-Becn1	GCGTCGGGGCCTAAAGAATG	CTCCTGGCTCTCTCCTGGTT	NM_053739.2
r-BiP	GACCACCTATTCCTGCGTCGGT	CGCCAATCAGACGCTCCCCT	NM_013083.2
r-Chop	AGGAGAGAGAAACCGGTCCAA	GGACACTGTCTCAAAGGCGA	NM_024134.2
r-Gapdh	ACAGCAACTCCCATTCTTCCA	TCCAGGGTTTCTTACTCCTTGG	NM_017008.4
r-Gnat1	TGACGTGCATCATTTTCATC	TTAAGCTCCAGGAACTGCAC	NM_001108780.2
r-Perk	GAAGTGGCAAGAGGAGATGG	GAGTGGCCAGTCTGTGCTTT	NM_031599.2
r-Rho	GTCCACTTCACCATCCCC	CTGCCTTCTGAGTGGTAGCC	NM_033441.1
m-Rho	ACAGTCAAGGAGGCGGCT	GCAAAGAAAGCTGGCAGAG	NM_145383.2

Table S3. Frequency of indels and frameshift in AAV-SaCas9/gRNA treated and untreated P23H rats.

Genotype	Groups	Number of Total Target Reads	Number of Reads with Indels	Frequency of Indels (%)	Number of Frameshift Reads	Frequency of Frameshift (%)
**P23H/P23H**	Untreated	44782	0	0.00	5	0.01
	AAV-treated	131250	6404	4.88	7596	5.78
**P23H/SD**	Untreated	50766	0	0.00	1	0.06
	AAV-treated	119481	13019	10.89	15032	12.58
**P23H/LE**	Untreated	42014	0	0.0	1	0.00
	AAV-treated	167609	21705	12.95	24375	14.54

Table S4. Off-target analysis of CRISPR/Cas9-mediated editing of *m-Rho*^{P23H} in P23H rats.

		**Off-Target 1**			**Off-Target 2**			**Off-Target 3**		
Genotype	Groups	Number of reads with indels	Number of total target reads	Frequency of Indels (%)	Number of reads with indels	Number of total target reads	Frequency of Indels (%)	Number of reads with indels	Number of total target reads	Frequency of Indels (%)
**P23H/P23H**	Untreated	0	149598	0	0	128268	0	0	141561	0
	AAV-treated	0	117695	0	0	102693	0	0	151268	0
**P23H/SD**	Untreated	0	103016	0	0	143373	0	0	153068	0
	AAV-treated	0	76721	0	0	136605	0	0	65796	0
**P23H/LE**	Untreated	0	55228	0	0	105284	0	0	201194	0
	AAV-treated	0	26572	0	0	133679	0	0	156138	0
